# PREVALENCE AND USE OF ADVANCED DRIVER ASSISTANCE SYSTEMS IN THE OLDER DRIVER POPULATION

**DOI:** 10.1093/geroni/igac059.2286

**Published:** 2022-12-20

**Authors:** Renee St Louis, David Eby, Lidia Kostyniuk, Lisa Molnar, Jennifer Zakrajsek, Nicole Zanier, Raymond Yung, Linda Nyquist

**Affiliations:** University of Michigan Transportation Research Institute, Ann Arbor, Michigan, United States; University of Michigan Transportation Research Institute, Ann Arbor, Michigan, United States; University of Michigan Transportation Research Institute, Ann Arbor, Michigan, United States; University of Michigan Transportation Research Institute, Ann Arbor, Michigan, United States; University of Michigan Transportation Research Institute, Ann Arbor, Michigan, United States; University of Michigan Transportation Research Institute, Ann Arbor, Michigan, United States; University of Michigan, Ann Arbor, Michigan, United States; University of Michigan, Ann Arbor, Michigan, United States

## Abstract

Research on advanced driver assistance systems (ADAS) in the older driver population has suggested the potential for ADAS to improve safety and driving comfort by helping aging drivers overcome functional declines commonly experienced in later-life. However, attaining anticipated ADAS benefits is dependent upon drivers’ awareness, understanding, and use of ADAS in their own vehicles. Questionnaire data from 2,374 older drivers enrolled in the AAA LongROAD study were analyzed to investigate changes in the prevalence and use of 15 ADAS and how participants learned to use these technologies. From baseline to Year 3, the prevalence of each ADAS significantly increased, with the greatest percentage point increase being for backup/parking assist technology (from 41.5% to 58.8%). The prevalence of one or more ADAS in participants’ vehicles increased from 59.0% to 72.0%, and the average number of ADAS per vehicle increased from 2.0 to 3.3. At both baseline and Year 3, approximately one-third of participants reported always using the ADAS available in their vehicle, but nearly one-quarter reported never using their ADAS. The largest proportion of participants at both baseline and Year 3 reported learning to use ADAS by figuring it out by themselves (45.5% and 50.8%, respectively), yet approximately 12.0% of participants at both time points reported never learning to use ADAS. To achieve the expected benefits of ADAS for older drivers, research is needed to better understand why ADAS are not being use more frequently when available, and to develop acceptable and accessible programs for training older adults to use ADAS.

